# Calcul géant compliquant un diverticule caliciel

**DOI:** 10.11604/pamj.2019.33.192.18077

**Published:** 2019-07-12

**Authors:** Mohammed Alae Touzani, Imad Ziouziou

**Affiliations:** 1 Service d’Urologie «B», Hôpital Avicenne, Rabat, Maroc; 2 Service d’Urologie, Hôpital Hassan II, Université Ibn Zohr, Agadir, Maroc

**Keywords:** Lithiase urinaire, traitement, malformation, diverticule caliciel, *Urinary lithiasis*, *treatment*, *malformation*, *calyceal diverticulum*

## Abstract

Calyceal diverticulum is acystic cavity in the renal parenchyma, communicating with the collecting system via an infundibulum. In more than 40% of cases it is associated with a stone and in most cases appears as milk of calcium forming a fluid level or multiple small stones. We here report the case of an 82-year old patient with no previous history, presenting with mild and intermittent right flank pain lasting for 8 years. Clinical examination was normal. The patient first underwent X-ray of urinary tree without preparation showing a density of calcium projecting into the upper pole of the right kidney (A). The patient then underwent uroscanner showing giant stone measuring 28mm within a caliciel diverticulum of the upper pole directly communicating with the renal pelvis (B). No associated urinary tract infection or haematuria were detected. Given patient's age and that he was paucisymptomatic and uninfected, therapeutic abstention and monitoring were indicated. In accordance with the recommendations of the experts, only symptomatic intradiverticular stones must be treated. First, extracorporeal shockwave lithotripsy (ESWL) allows improvement to the symptoms in 1 patient out of 2 and no stone fragmentation in 1 patient out of 4. In second intention, flexible uretero-renoscopy should be performed. Percutaneous nephrolithotomy can be performed if the stone is located in the lower pole (rare). Finally, in case of failure, laparoscopic treatment or open surgery may be proposed.

## Image en médecine

Le diverticule caliciel correspond à une cavité kystique dans le parenchyme rénal, communiquant avec le système collecteur via un infundibulum. Il s'y associe dans plus de 40% des cas un calcul et prend le plus souvent un aspect de niveau hydrique de tonalité calcique, ou de multiples calculs de petite taille. Nous rapportons ici le cas d'un patient de 82 ans, sans antécédents personnels, souffrant de douleurs légères et intermittentes au flanc droit depuis 8 ans. L'examen clinique était normal. Le patient a d'abord bénéficié d'une radiographie de type arbre urinaire sans préparation montrant une image de tonalité calcique se projetant dans le pôle supérieur du rein droit (A). Le patient a ensuite bénéficié d'un uroscanner montrant un calcul géant de 28mm dans un diverticule caliciel du pole supérieur communiquant directement avec le pelvis rénal (B). Il n'y avait pas d'infection urinaire associée, ni d'hématurie. Étant donné que le patient était pauci-symptomatique, non infecté et compte tenu de son âge, nous avons décidé l'abstention thérapeutique et la surveillance. Conformément aux recommandations des experts, seuls les calculs intra-diverticulaires symptomatiques doivent être traités. En premier lieu, la LEC permet une amélioration de la symptomatologie chez 1 patient sur 2 et un résultat sans fragment chez 1 patient sur 4. En deuxième intention, une urétéro-rénoscopie souple a tout son intérêt. En cas de localisation inférieure de la pierre, ce qui est rare, une NLPC peut être proposée. Enfin, en cas d'échec, un traitement par laparoscopie ou chirurgie ouverte peut être proposé.

**Figure 1:**
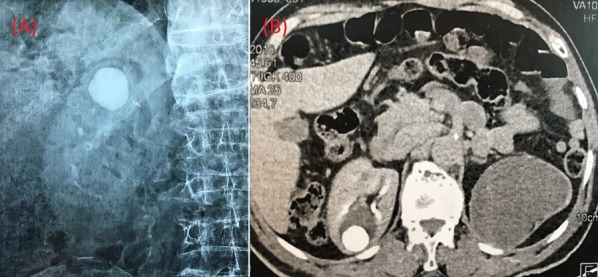
(A) arbre urinaire sans préparation; (B) uroscanner-coupe transversale

